# Retrospective evaluation of postoperative joint immobilization using a temporary calcaneotibial screw for medial or lateral tarsocrural joint instability in dogs

**DOI:** 10.1111/vsu.14081

**Published:** 2024-03-08

**Authors:** Yuya Saitoh, Andrew J. Worth, Hugh Hasselman, Sacha Devereux

**Affiliations:** ^1^ School of Veterinary Science Massey University Palmerston North New Zealand; ^2^ VetSouth Gore New Zealand

## Abstract

**Objective:**

To evaluate the use of a temporary calcaneotibial screw (CTS) to immobilize medial or lateral tarsocrural joint instability (TCI) in dogs.

**Study design:**

Retrospective study.

**Animals:**

Twelve dogs (including five active working farm dogs) with TCI.

**Methods:**

Medical records (January 2015–June 2023) were retrospectively reviewed for cases of TCI managed surgically including temporary joint immobilization using a CTS and external coaptation. Clinical data consisted of medical records and an online survey completed by the owner.

**Results:**

Surgical techniques to address TCI included primary ligamentous repair, synthetic ligament reconstruction, or malleolar fracture repair. Immobilization with a CTS was employed for 6–8 weeks postoperatively. The online survey was completed for 10 dogs. All dogs exhibited good‐to‐excellent functional outcomes at the follow‐up (median, 31 months; range, 4–66). All working farm dogs (5) were able to return to normal or substantial levels of their work. Four distinct complications were reported in three dogs including one CTS breakage and three bandage‐related soft‐tissue injuries.

**Conclusion:**

This retrospective study represents the first report of employing a temporary CTS for TCI in dogs.

**Clinical significance:**

A temporary CTS was effective in immobilizing the tarsocrural joint for dogs with TCI and the postoperative complication rate in this study was relatively low. A CTS screw and external coaptation is a viable alternative to previously reported methods of tarsocrural joint stabilization.

## 1 INTRODUCTION

The medial (tibial) and lateral (fibular) collateral ligaments provide medial and lateral support for the tarsocrural joint, respectively.^1^, ^2^ In dogs, each tarsocrural collateral ligament complex is comprised of three distinct ligaments: one long and two short.^1^ Medial or lateral tarsocrural joint instability (TCI) is associated with complete or partial tarsal collateral ligament ruptures, malleolar fractures at the origins of the ligaments on the tibia or fibula, or shearing injuries.^1^, ^2^, ^3^, ^4^, ^5^, ^6^ TCI is typically secondary to trauma and is associated with varying degrees of hindlimb lameness. While the extent and direction of dislocation may vary, disruption of the medial ligaments is much more common, attributed to the slight valgus posture of the tarsal joint.^1^, ^3^ Especially in sheep and beef cattle farming areas, TCI is frequently diagnosed in active working farm dogs, renowned for swiftly maneuvering to guide and control livestock, due to encounters with livestock, automobiles, and fences.^7^, ^8^

Surgical treatment for the complete or partial collateral ligament rupture includes primary ligamentous repair^1^, ^2^, ^4^, ^6^ and synthetic reconstruction/reinforcement.^1^, ^2^, ^3^, ^4^, ^5^, ^9^, ^10^ The necessity for malleolar fracture repair^1^, ^2^, ^3^, ^4^, ^6^ and open wound management of shearing injuries^1^, ^2^, ^3^, ^4^ is contingent upon the nature and severity of the injury. Although primary repair of the ligament is not always possible, it should be attempted because restoring the continuity of the ligament can optimize healing.^1^, ^2^, ^4^, ^6^ Synthetic ligament reconstruction can also provide joint stability when primary repair is impossible or inadequate. The reconstruction can be performed using bone anchors, screws with washers, or bone tunnels as anchor points for nonabsorbable sutures/prostheses to augment or replace the ligament.^1^, ^2^, ^3^, ^4^, ^5^, ^9^, ^10^ Most malleolar fractures can be repaired with a pin and tension band wire.^1^, ^2^, ^3^, ^4^, ^6^ Shearing injuries with extensive soft tissue damage may necessitate open wound management.^1^, ^2^, ^3^, ^4^

However, all the aforementioned techniques will be too weak to allow physiological forces until healing occurs therefore an TCI repairs must be supported/immobilized postoperatively to avoid treatment failures.^3^ Temporary immobilization is advocated for a minimum of 4–6 weeks following ligament repair or reconstruction, assisting in the healing process or fibrous tissue replacement of the ligaments.^1^, ^2^, ^3^, ^5^, ^6^ The use of transarticular external skeletal fixation (ESF) or external coaptation (EC) with or without a splint/cast are the most commonly reported.^1^, ^2^, ^3^, ^4^, ^5^, ^6^, ^9^, ^10^ Transarticular ESF is generally favored for cases involving open wound management and by many surgeons for cases suitable for primary repair.^3^, ^4^, ^11^ Although temporary immobilization is considered necessary for TCI due to a high incidence of postoperative implant failures,^3^ it is also known that most complications of the management of tarsal instability are related to postoperative temporary immobilization.^1^, ^4^, ^11^, ^12^ In some cases with no hope of preserving a functional joint such as highly comminuted fracture or failure of the initial repair, pantarsal arthrodesis or amputation may be considered as a salvage procedure.^1^, ^2^, ^4^, ^8^

The calcaneotibial screw (CTS) is an effective method of temporarily immobilizing the tarsocrural joint in extension following the repair of the common calcanean (Achilles) tendon ruptures in dogs.^13^, ^14^, ^15^ An ex vivo study^16^ has demonstrated that a CTS restores medial and lateral stability to the tarsocrural joint after collateral ligament transection, comparable to intact ligaments. When used to support common calcanean tendon repairs, a temporary CTS is typically combined with EC to mitigate issues such as cyclic loading of the screw and premature screw breakage,^1^, ^2^, ^13^, ^15^ and it is speculated that this combination would result in fewer bandage‐related complications due to the elimination of joint motion within the EC.^13^

On the basis of our previous cadaveric study,^16^ this clinical case study was undertaken to document the outcomes of surgical intervention for TCI in consecutive dogs, each supported postoperatively with a CTS and EC. We hypothesized that a temporary CTS would provide effective postoperative immobilization of the tarsocrural joint even in active working farm dogs and have a lower complication rate compared to previous reports.

## 2 MATERIALS AND METHODS

Medical records of all dogs diagnosed with TCI that underwent surgical stabilization between January 2015 and June 2023 at the Massey University Veterinary Teaching Hospital, Palmerston North, New Zealand, and VetSouth, Gore, New Zealand were reviewed. The diagnosis of TCI and concurrent injury was established on the basis of clinical examination supported by radiographic assessment including stress‐applied radiographs,^1^, ^6^ and confirmed by intraoperative findings. Signalment, bodyweight, initiating cause, affected limb, duration of lameness, detail of TCI (medial, lateral, or bilateral collateral ligament rupture, malleolar fracture, and shearing injury), concurrent injuries, surgical procedures, postoperative temporary immobilization technique, osteoarthritis (OA), and complications were collected for all dogs. Outcome data was also collected from owners using a web‐based online survey administration software (Google Forms, Google) and included the current status of the dog, pre‐ and postoperative lameness scores, any complications, and the subjective owner satisfaction with the outcome of the surgery and the financial investment [https://forms.gle/LphfB9fBTjZoofo49]. The degree of lameness experienced by the dogs before and after the treatment was evaluated by the same owner using a numerical scale ranging from 0 to 5 (0 = unaffected; 1 = constant gait abnormality, mild intermittent lameness; 2 = constant gait abnormality, mild persistent lameness; 3 = constant gait abnormality, moderate, persistent lameness, intermittent nonweight‐bearing; 4 = constant gait abnormality, severe, persistent lameness, nonweight‐bearing at walk/trot; 5 = nonweight‐bearing even at rest). The owners were requested to rate their overall level of satisfaction on a scale ranging from 1 to 5 (1 = being very disappointed to 5 = being very satisfied). For owners of active working farm dogs, additional inquiries were made regarding their type of farming system (dairy, intensive sheep/beef, extensive sheep/beef, or other), work environments (flat, rolling hill, hill country, or hard hill country), and roles (heading, huntaway, yarding, breeding, or retiring). Additionally, the owners of working farm dogs provided ratings regarding the extent of their dogs' return to work, employing a scale ranging from 1 to 5 (1 = can perform normal duties; 2 = can perform most duties, but allowances have to be made; 3 = can perform some duties but is of limited usefulness; 4 = can perform few duties, very limited usefulness; 5 = not useful as a working farm dog).^13^ If postoperative complications were reported by owners and the dog was managed at other veterinary hospitals, the treating veterinarian's information was sought to obtain details of the complication and treatment. For cases that did not respond to the survey, as much information as possible was collected from the medical records of the primary and referral hospitals. The same lameness scale was used for the cases, but the ratings were made by veterinarians rather than owners. Cases included in this study met the criteria of undergoing surgical treatment for TCI.

## 3 RESULTS

Twelve dogs with TCI were included in the study (Table 1). At surgery, the median age of the dogs was 3.5 years (mean 5.3 years, range 1–14 years), and their median bodyweight was 30.0 kg (mean 28.4 kg, range 21.7–35.0 kg). There were six males (five intact) and six females (two intact). Five of the 12 dogs were active working farm dogs, including two NZ Heading dogs and three NZ Huntaways. The five dogs were employed on intensive (1) or extensive (4) sheep and/or beef‐type farms situated in hill countries. The traumatic events leading to TCI involved five livestock‐associated injuries (with four cattle and one horse), four automotive accidents, two jumping injuries, and one entrapment in the fence. All TCI were unilateral: affecting eight right and four left pelvic limbs, while involving nine medial and three lateral instabilities including two lateral malleolar fractures and two shearing injuries of the tarsus. Eleven dogs had complete or partial collateral ligament rupture including one dog with ligament rupture and a malleolar fracture (Case 6), whereas one dog had a malleolar fracture with intact ligaments (Case 7). Concurrent injuries included a complete simple tibial‐fibular diaphyseal fracture and a lateral instability at the calcaneoquartal joint. The traumatic events had occurred a median of 5 days (mean 11.3 days, range 1–51 days) prior to surgery.

**TABLE 1:** Medial or lateral tarsocrural joint instability treated by calcaneotibial screw and external coaptation in 12 dogs.

Case	Signalment	Initiating cause and duration of lameness prior to surgery	TCI and concurrent injury	Surgical procedures and temporary immobilization	Pre‐ and postoperative LMS^a^	Follow‐up period and complications	Owner satisfactions^b^ Question 1 Question 2 Level of satisfaction	Working type and scale of the return to work^c^
1	3‐yr‐old 27.6 kg Female NZ Huntaway	Livestock 1 d	Left‐medial	PLRP 4.5 mm–56 mm CTS and BC	5 → 0	35 mo	Yes Yes 4/5	Hill country Intensive Huntaway 1/5
2	4‐yr‐old 29.2 kg Male NZ Heading dog	Livestock 7 d	Right‐lateral	SLRC with screw and bone tunnel 4.5 mm–60 mm CTS and BC	2 → 1	42 mo	Yes Yes 5/5	Hill country Extensive Heading 1/5
3	9‐yr‐old 22.3 kg Male NZ Heading dog	Livestock 3 d	Right‐medial	SLRC with screw and bone tunnel 4.5 mm–56 mm CTS and BC	5 → 1	66 mo	Yes Yes 5/5	Hill country Extensive Heading 2/5
4	4‐yr‐old 31.2 kg Female NZ Huntaway	Fence 2 d	Right‐lateral	SLRC with screw and bone tunnel 3.5 mm–60 mm CTS and CS	5 → 0	35 mo^d^	Yes Yes 5/5	Hill country Extensive Huntaway 1/5
5	3‐yr‐old 35.0 kg Male NZ Huntaway	Livestock 9 d	Right‐medial	SLRC with screw and bone tunnel 4.5 mm–50 mm CTS and CS	5 → 1	12 mo^d^	Yes No Due to the short working period to the second accident 4/5	Hill country Extensive Huntaway 2/5
6	8‐yr‐old 31.5 kg Spayed female Labrador Retriever	Jumping 1 d	Right‐medial with lateral MFx	SLRC and MFxR with lag screw and bone tunnel 4.5 mm–50 mm CTS and BC	5 → 2	47 mo	Yes Yes 4/5	–
7	3‐yr‐old 32.0 kg Castrated male Labrador Retriever	Jumping 7 d	Right‐lateral with lateral MFx CQI	MFxR with Kirschner wire and surgical wire A short plate for the CQI 3.5 mm–50 mm CTS and CS	4 → 1	4 mo CTS breakage and BSTI	Yes Yes 2/5 Due to the cost for complication treatment	–
8	1‐yr‐old 30.9 kg Male Labrador Retriever	Automotive 22 d	Left‐medial with SI	SLRC with screw 3.5 mm–45 mm CTS and CS	5 → 1	6 mo	Yes Yes 4/5	–
9	2‐yr‐old 25.4 kg Male NZ Huntaway	Livestock 28 d	Left‐medial	SLRC with screw 3.5 mm–50 mm CTS and CS	5 → 1	27 mo BSTI	Yes Yes 5/5	–
10	14‐yr‐old 32.0 kg Spayed female Labrador Retriever	Automotive 2 d	Right‐medial with SI T/F Fx	PLRP DCP and hemi‐cerclage wire for the T/F Fx 3.5 mm–45 mm CTS and CS	5 → 2	24 mo^d^	Yes Yes 5/5	–
11	3‐yr‐old 21.7 kg Spayed female Bull Terrier	Automotive 51 d	Right‐medial	PLRP 3.5 mm–50 mm CTS and CS	(2 → ‐)	(1 mo)	–	–
12	9‐yr‐old 22.0 kg Spayed female Labrador Retriever	Automotive 3 d	Left‐medial	SLRC with screw 3.5 mm–45 mm CTS and CS	(5 → 0)	(39 mo) BSTI	–	–

Abbreviations: BC, bivalve cast; BSTI, bandage‐related soft‐tissue injury; CQI, calcaneoquartal joint instability; CS, cranial splint; CTS, calcaneotibial screw; DCP, dynamic compression plate; Fx, fracture; LMS, lameness score; MFxR, malleolar Fx repair; mo, month; NZ, New Zealand; PLRP, primary ligamentous repair; SI, shearing injury; SLRC, synthetic ligament reconstruction; T/F Fx, tibial‐fibular Fx; TCI, tarsocrural joint instability (medial or lateral); yr, year.

^a^ Pre‐ and postoperative lameness were scored by the same owner on a scale of 0–5 (0 = unaffected; 1 = constant gait abnormality, mild intermittent lameness; 2 = constant gait abnormality, mild persistent lameness; 3 = constant gait abnormality, moderate, persistent lameness, intermittent nonweight‐bearing; 4 = constant gait abnormality, severe, persistent lameness, nonweight‐bearing at walk/trot; 5 = nonweight‐bearing even at rest).

^b^ Two yes/no questions and overall satisfaction level were asked. Q1: Did the result of the surgery meet your expectations? Q2: Was the financial investment for surgery worthwhile? The level of overall satisfaction was on a scale of 1–5 (1 = being very disappointed to 5 = being very satisfied).

^c^ The working farm dog's owners scored the level of return to work on a scale of 1–5 (1 = can perform normal duties; 2 = can perform most duties, but allowances have to be made; 3 = can perform some duties but is of limited usefulness; 4 = can perform few duties, very limited usefulness; 5 = not useful as a working farm dog).

^d^ Euthanasia for reasons different from this treatment.

**FIGURE 1:**
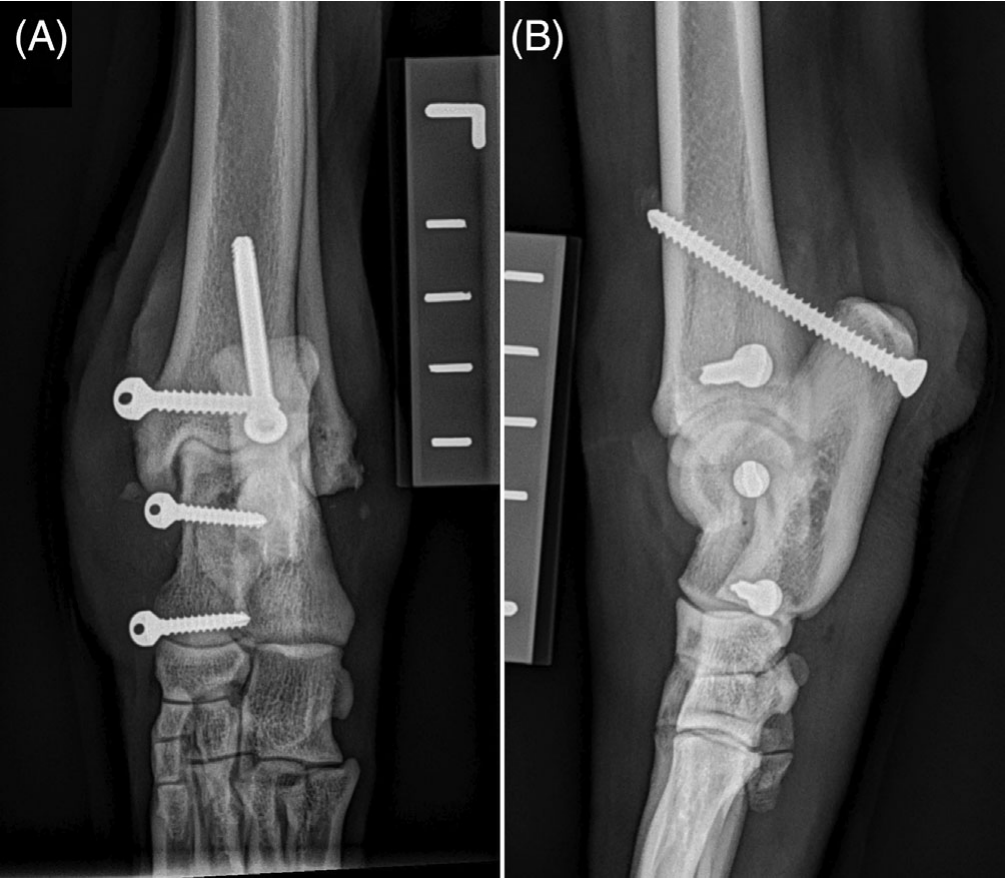
Postoperative craniocaudal (A) and mediolateral (B) radiographs of Case 8. The synthetic ligament reconstruction using three bone anchors was performed in the dog. A calcaneotibial screw (CTS) was placed at an angle perpendicular to the long axis of the calcaneus with the tarsus in full extension.

**FIGURE 2:**
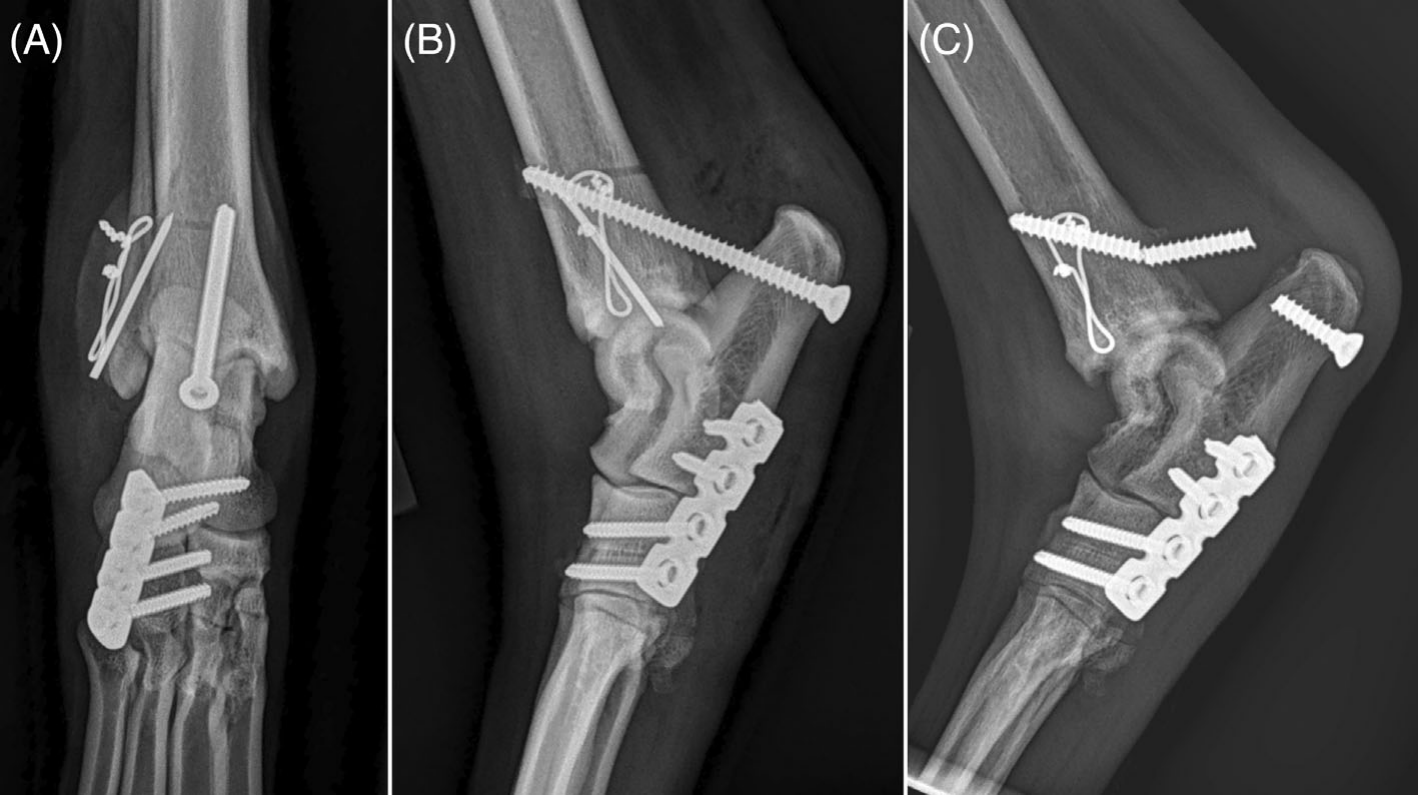
Postoperative craniocaudal (A) and mediolateral (B) radiographs of Case 7. The malleolar fracture was repaired with a Kirschner wire and a surgical wire. To stabilize the lateral instability at the calcaneoquartal joint, a short plate and screws were utilized. The calcaneotibial screw (CTS) breakage happened 8 weeks after surgery (C).

All 12 dogs underwent surgical treatment of TCI followed by CTS and EC. Primary collateral ligament repair using a monofilament absorbable suture (polydioxanone suture) in a locking loop or figure‐eight suture pattern was performed in three dogs (Cases 1, 10, and 11). In eight dogs (Cases 2–6, 8, 9, and 12) ligament repair was impossible or deemed inadequate therefore synthetic ligament reconstruction using bone anchors (Figure 1), screws with washers, or bone tunnels was performed. Two malleolar fractures (Cases 6 and 7) were repaired with a lag screw technique or Kirschner wire and tension band wire (Figure 2A,B). In Case 6, synthetic ligament reconstruction was simultaneously performed because of the concomitant collateral ligament rupture and malleolar fracture. A CTS (Figure 1) was placed with the tarsocrural joint held in extension using reduction forceps. The calcaneus and tibia were drilled and tapped for a positional cortical screw which varied in size from 3.5 to 4.5 mm in diameter depending on the size of the dog. Care was taken to direct the pilot hole proximally, to avoid entering the tarsocrural joint, and medially in order to penetrate the center of the tibia. The positional screw was placed approximately perpendicular to the long axis of the calcaneus and its head was not countersunk.^13^, ^14^, ^16^ For the tibial fracture (Case 10), a dynamic compression plate and an additional hemi‐cerclage wire were used. To stabilize concurrent lateral instability at the calcaneoquartal joint (Case 7), a short plate and screws were utilized (Figure 2A,B). Primary closure of all surgical incisions was possible, even for dogs involving shearing injuries (Cases 8 and 10). No open wound management was therefore necessary. Postoperative radiographs were taken to ascertain the correct positioning of the implants.

The hind limb was then subsequently supported in a cranial splint (Cases 4, 5, and 7–12) or a bivalve cast (Cases 1–3, and 6).^8^, ^13^, ^15^ This difference depended on the routines of the two hospitals. The owners were instructed to house the dogs in a dry and sheltered kennel with no exercise. The EC was to be checked weekly by veterinarians and daily by owners, and the dressing was changed as required. Five dogs (Cases 5, 7–9, and 10) were transferred back to their original or local general practices for dressing changes. At 6–8 weeks after surgery, the CTS was removed under a short general anesthetic. Following the removal of the screw, a Robert‐Jones bandage (Cases 4, 5, and 7–12) or a cranial splint (Cases 1–3, and 6) was applied for further 1–2 weeks. Owners were instructed to allow only limited leash‐controlled exercise for 4–6 weeks subsequent to the removal of the screw. For working farm dogs, light work duties were allowed from 12 to 16 weeks, with normal work levels allowed at 16–20 weeks postoperatively.

Follow‐up information via the online survey was available for 10 dogs. In the other two dogs (Cases 11 and 12), the owners could not be contacted. The median duration of the follow‐up period was 31 months (mean 29.8 months, range 4–66 months). For dogs that had died before the survey commenced, the follow‐up interval was defined as the time elapsed between the surgery and euthanasia. Three dogs had already been euthanized at the time the survey was conducted, due to pleural effusion (Case 4), traumatic on‐farm injury (Case 5), and automotive accident (Case 10). As the owner‐evaluated preoperative lameness score, eight dogs scored 5/5 and one dog scored 4/5, the remaining one dog experienced mild persistent lameness with a score of 2/5. For Cases 11 and 12 (no owner score), preoperative lameness was scored 2/5 and 5/5, respectively by veterinarians. In all 10 dogs, there was a resolution or improvement of lameness. The lameness score at follow‐up was reported as 0/5 in two dogs, 1/5 in six dogs, and 2/5 in two dogs. In the other two cases (Case 11 and 12) without the survey, information was collected from medical records. The last recheck in Case 11 was performed a month postoperatively until the removal of CTS, and subsequent follow‐up data was not available. Case 12 visited the hospital 39 months postoperatively and the veterinarian‐evaluated lameness score at that time was 0/5. All working farm dogs (5) could return to their work and could perform most duties: 1/5 in three dogs and 2/5 in two dogs. Although not all dogs underwent long‐term follow‐up radiographs, the clinical records revealed the diagnosis of OA within the surgically treated tarsal joint in three dogs (Cases 3, 6, and 9), warranting the administration of nonsteroidal anti‐inflammatory drugs for the amelioration of their OA throughout the follow‐up period.

Complications were reported in three dogs: four distinctive complications including one major complication (a CTS breakage) and three minor complications (bandage‐related soft‐tissue injuries). Case 7 experienced a CTS breakage and a bandage‐related soft‐tissue injury requiring antibiotic medication for 2 weeks. Both were incidentally diagnosed at the scheduled date of screw removal 8 weeks postoperatively. The referring veterinarian reported that the splint had not been rechecked for more than 5 weeks, due to a lack of owner compliance with postoperative instructions. Cases 9 and 12 also experienced bandage‐related soft‐tissue injuries requiring antibiotic medication for 2 weeks while the CTS was in place. Two (Cases 7 and 9) out of the three dogs with soft‐tissue injuries were not using E‐collars at home at the discretion of their owners and had records of chewing and licking the bandage. All complications were successfully resolved within 2 weeks.

The 10 owners who completed the survey expressed satisfaction with the outcome of the surgery and affirmed that their expectations were met. Nine owners answered that the financial investment for the surgery was worthwhile, whilst the owner of a working farm dog (Case 5), which was euthanized due to another traumatic on‐farm injury that occurred 12 months after the surgery, answered in the negative. For their overall level of satisfaction, five owners rated 5/5, four rated 4/5, and one rated 2/5. The last owner (Case 7) explained that it was because of the additional cost of the treatment for complications.

## 4 DISCUSSION

This retrospective study demonstrates that temporary tarsocrural immobilization using a CTS and EC can be used to support primary surgical treatment of TCI in dogs. All 10 dogs with complete follow‐up were successfully managed. Specifically, all dogs had resolution or improvement of lameness following the treatment without any reported treatment failures resulting in instability. Whilst subjective and biased by personal circumstances, the high ratings for owner satisfaction and whether the procedure was financially worthwhile were useful as part of a prospective evaluation of a new technique.

Our decision to investigate the effectiveness of CTS to immobilize the tarsocrural joint in TCI was primarily motivated by three factors: its relative ease of application and postoperative management, its cost‐effectiveness, and its potential to reduce complications. A previous ex vivo study^16^ suggested that CTS fixation would be an adequate method of stabilizing the tarsocrural joint following TCI in clinical cases. The results consistently demonstrated that the CTS‐stabilized limb, regardless of the variation of collateral ligament transection, exhibited stability comparable to the uninjured limb in both varus and valgus stress.

TCI is common in sheep and beef cattle farming areas, with their unique environment and the high number of working farm dogs.^7^, ^8^ Five of the 12 dogs reported were active working farm dogs and the mechanisms of their TCI were similar to those reported in the previous studies. When selecting a TCI treatment method, the practicality of postoperative management is a factor for veterinarians/owners in rural practices. The distance between farms and surgical referral centers can make the choice of transarticular ESF and its requirement for expert monitoring problematic. In this study, five dogs were transferred back to their original or local general practices for dressing changes as the owners chose the closer option due to the distance from their homes or farms to our referral centers. The technique described in this study is also likely to be cheaper to perform than a transarticular ESF since it requires only one screw (plus EC) for immobilization. One study indicated that ESF had a longer surgical time and higher total costs compared to the EC group following calcaneal tendon rupture repair.^11^ Cost‐effectiveness is particularly important when treating working farm dogs in comparison to pets.^8^, ^13^

Lameness scores assessed by the owners were utilized in this study as a means of evaluating pre‐ and postoperative conditions of gait. Despite their subjective nature, owner assessments have been recognized as of value for demonstrating postoperative outcomes.^8^, ^13^ All 10 dogs showed resolution or improvement in lameness, however persistent mild lameness (2/5) remained in two dogs (Cases 6 and 10) likely due to the extent of the trauma and joint injury not referable to the CTS screw. Different from common calcaneal tendon rupture repair, OA is a likely sequela following TCI as the injury and method of repair may compromise the joint.^3^, ^6^, ^13^ The majority of our owners, however, expressed satisfaction regardless of any remaining lameness. In active working farm dogs, additional factors, such as financial outlay, time off work, level of work, and potential years before the progression of OA, may influence the owners' satisfaction levels. We propose that assessing the dog's functional recovery in relation to its ability to resume work should be the primary measure of outcome in working farm dogs.^8^, ^13^

In this study, complications occurred in three limbs, with one joint exhibiting two complications, resulting in a total of four distinct complications (one major and three minor) that do not necessitate implant (other than intended CTS) removal or revision of the ligament repair/reconstruction. In a study^3^ that did not use postoperative temporary immobilization in cases treated by synthetic ligament reconstruction, major complications requiring implant removal were observed in 43.8% (seven of 16 dogs) including four infections, loosening of two implants sufficient to cause discomfort, and one broken wire causing lameness. Previous studies of surgical stabilization of canine tarsal joints have reported varying rates of complications, ranging up to 92%.^4^, ^11^, ^12^, ^13^, ^15^ The consensus among these reports is that complications generally stem from methods of temporary immobilization rather than the surgical repair itself. These methods for the tarsocrural joint can be classified into four main categories: transarticular ESF, CTS, EC, or a combination of the aforementioned techniques. According to previous studies, major complications resulting from EC appear to be less prevalent compared to ESF; however, minor complications are not infrequent.^4^, ^11^, ^12^, ^15^ In one report, 63% of 60 animals (38 pelvic limbs) treated with EC developed soft‐tissue injuries (60% mild, 20% moderate, and 20% severe).^12^ The complications observed in our study appear to be relatively few compared to the previous reports^4^, ^11^, ^12^ with immobilization procedures including ESF and EC that did not utilize CTS in most cases.

When CTS was combined with EC, major complications were observed in 10% (1 of 10) of cases in our study and 14.3% (1 of 7) of cases with common calcaneal tendon ruptures.^13^ In addition, minor bandage‐related complications in our study were identified in 27% (3 of 11) cases. Worth et al. described 10 active working farm dogs that underwent surgical treatment of common calcaneal tendon rupture using CTS in combination with an EC (7) or EC only (3) and reported that dogs only supported with an EC tended to have poorer outcomes.^13^ Combining CTS with coaptation techniques may yield better outcomes by eliminating joint motion within the splint thus reducing skin abrasion. Although some surgeons have successfully used a CTS without any external support,^1^ it has been advised that EC is used in combination with a CTS to avoid cyclic loading of the screw and premature screw breakage, especially in large dogs.^16^ The screw should remain in position until the tendon repair is in the proliferation and remodeling phases, even after which they need some external support and activity restriction. In our study, all complications were successfully managed and did not affect the overall outcome, although one owner expressed disappointment due to additional treatment costs stemming from the removal of a broken CTS. Premature loosening or breakage of CTS can occur due to high axial loading force and cyclic loading, necessitating surgical replacement or early removal of the screw. In a study of 38 dogs in which CTS was used, three complications associated with broken or bent screws were reported.^15^ In the one case of CTS breakage in our study, the hock was more flexed than ideal and the joint angle may have increased the load on the screw (Figure 2C). This was the dog with poor owner compliance, and had the problem been identified at scheduled checks the complications might have been mitigated. Two of three dogs with minor bandage‐related complications had a history of licking and chewing on the bandage. This was thought to be due to irritation or pressure sore associated with the bandage. To minimize complications, it is crucial to adhere to proper CTS placement, conduct weekly bandage rechecks by a veterinarian, and closely monitor clinical signs related to the temporary immobilization.

Limitations of this study primarily stem from its retrospective nature and relatively small sample size. The medial and lateral TCI were not grouped, and there was variability in the injuries. Consequently, divergences emerged in surgical procedures. While some studies have explored the characteristics of synthetic ligaments for tarsal collateral ligament ruptures,^5^, ^10^ the prognostic variances contingent upon the type of syntheses and repair techniques remain incompletely elucidated. Also, a detailed assessment of rupture in each of the three distinct parts of the collateral ligament was omitted due to incomplete clinical records and the impracticality of precise grading of their individual severities. In two cases, the subjective assessment of complications and prognosis should be cautiously interpreted because of the lack of an owner's assessment and incomplete follow‐up. Also, it would have been beneficial to evaluate the progression of OA on images at different time points after surgery.

In conclusion, a temporary CTS with EC resulted in a good outcome when used as the means of temporary immobilization for surgical management of TCI in 10 dogs including five working farm dogs. Although persistent lameness may remain in some cases and OA may subsequently develop, dogs can return to good function and working farm dogs can return to work. This study observed a relatively low rate of postoperative complications in comparison to previously reported methods of tarsocrural joint stabilization.

## AUTHOR CONTRIBUTIONS

Saitoh Y, BVSc: Conceptualization and design of the study, drafting the manuscript, data collection, data interpretation, and agreement to be accountable for all aspects of the study in ensuring that questions related to the accuracy or integrity of any part of the work are appropriately investigated and resolved; Worth AJ, BVSc, PhD, FANZCVS (Small Animal Surgery): Conceptualization and design of the study, data collection, data interpretation, and revising the manuscript; Hasselman H, BVSc: Design of the work, data collection, and revising the manuscript; Devereux S, MVB, DECVS: Design of the work, data collection, and revising the manuscript. All authors provided a critical review of the manuscript and endorsed the final version, and are aware of their respective contributions and have confidence in the integrity of all contributions.

## CONFLICT OF INTEREST STATEMENT

The authors declare no conflict of interest related to this report.
